# Adipose Mesenchymal Stem Cell Metabolites Oral Gel Enhance Pro-Angiogenic Factors Expression, Angiogenesis, and Clinical Outcome of Oral Ulcer Rat Model

**DOI:** 10.1055/s-0043-1761192

**Published:** 2023-03-24

**Authors:** Satutya Wicaksono, Alexander Patera Nugraha, Jola Rahmahani, Fedik Abdul Rantam, Suryo Kuncorojakti, Helen Susilowati, Wibi Riawan, Ira Arundina, Pudji Lestari, Resgita Nadila Masya, Meircurius Dwi Condro Surboyo, Diah Savitri Ernawati

**Affiliations:** 1 Master Program in Immunology, Postgraduate School, Universitas Airlangga, Surabaya, Indonesia; 2 Department of Orthodontics, Faculty of Dental Medicine, Universitas Airlangga, Surabaya, Indonesia; 3 Division of Veterinary Microbiology, Department of Veterinary Science, Faculty of Veterinary Medicine, Universitas Airlangga, Surabaya, Indonesia; 4 Division of Veterinary Anatomy, Department of Veterinary Science, Faculty of Veterinary Medicine, Universitas Airlangga, Surabaya, Indonesia; 5 Research Center for Vaccine Technology and Development, Institute of Tropical Disease, Universitas Airlangga, Surabaya, Indonesia; 6 Department of Biomolecular Biochemistry, Universitas Brawijaya, Malang, Indonesia; 7 Department of Oral Biology, Faculty of Dental Medicine, Universitas Airlangga, Surabaya, Indonesia; 8 Department of Public Health and Preventive Medicine, Faculty of Medicine, Universitas Airlangga, Surabaya, Indonesia; 9 Graduate Program in Dental Health Science, Faculty of Dental Medicine, Universitas Airlangga, Surabaya, Indonesia; 10 Department of Oral Medicine, Faculty of Dental Medicine, Universitas Airlangga, Surabaya, Indonesia

**Keywords:** oral ulcer, regenerative medicine, mesenchymal stem cell, dentistry

## Abstract

**Objective** Enhancing wound healing capacity is one of the main principles in oral ulcer management. Efficient oral ulcer management will accelerate clinical symptom amelioration and prevent complications. Adipose mesenchymal stem cell metabolites (AdMSCM), a novel biological product, contains a plethora of bioactive mediators that can induce a series of processes in wound healing. This study will analyze the clinical outcome, angiogenesis, and expression of FGF-2 and VEGFA in the oral ulcer rat model after AdMSCM oral gel application.

**Materials and Methods** Twenty healthy male Wistar rats ( *Rattus novergicus* ) were used to create oral ulcer animal models. AdMSCM oral gel treatment was performed three times daily for 3 and 7 days. Clinical outcome was assessed by measuring the major diameter of the ulcer; the angiogenesis was evaluated through histological assessment; the expression of VEGFA and FGF-2 was assessed using the immunohistochemistry method.

**Statistical Analysis** This study uses parametric comparative analysis using one-way analysis of variance (ANOVA) and post-hoc Tukey's HSD test

**Results** The application of AdMSCM oral gel in an oral ulcer rat model significantly enhanced the clinical outcome ( *p* < 0.05). In addition, similar results were shown in the histologic assessment of angiogenesis and supported by the significant increase of VEGFA and FGF-2 expression.

**Conclusions** AdMSCM oral gel accelerates oral ulcer healing processes, proven by the enhancement of angiogenesis, pro-angiogenic factors expression, and clinical outcomes.

## Introduction

Accelerating the oral ulcer healing process is pivotal in managing pain and discomfort and preventing complications. 1 The healing process of oral ulcers is a complex mechanism that involves various cellular and molecular components. The ulcer healing mechanism consists of four overlapping spatiotemporal phases: hemostasis, inflammation, proliferation, and remodeling. 2 Under normal conditions, the healing period for oral mucosal ulcers is between 7 and 14 days. Still, several local (e.g., local irritants, infection) and systemic factors (e.g., diabetes mellitus, aging, vascular insufficiencies, medication) may impede the healing process. 2 3 One of the essential aspects of the healing process is angiogenesis. Angiogenesis is the mechanism of capillaries formation from pre-existing blood vessels. This biological event is pivotal in wound healing because it facilitates adequate blood and nutrient supply and transports many cells and molecular factors to the wound site. The previous study has also reported that insufficient angiogenesis may lead to chronic, non-healing wound formation. The initiation of angiogenesis requires the stimulation of several pro-angiogenic factors, such as vascular endothelial growth factor (VEGF) and fibroblast growth factor-2 (FGF-2). 2 3 4

The current pharmacological approaches for oral ulcers (i.e., topical steroid, non-steroid, and herbal medication therapies) still have several clinical limitations in their use, especially when it comes to managing chronic or extensive oral ulcer cases. For instance, inappropriate timing and dosage of topical corticosteroid drugs can inhibit wound healing due to their anti-inflammatory and anti-mitotic effects. 5 6 Karavana-Hizarcioğlu et al also identified that the healing rate of oral ulcers treated with benzydamine hydrochloride only differed by 33% compared with the placebo group. 7

Constant scientific developments, especially in regenerative medicine, have induced the discovery of several therapeutic biological products. Mesenchymal stem cells (MSCs) are one of the most notorious technologies in regenerative medicine that have been widely explored. 8 The recent development of MSCs is utilizing their culture medium, previously regarded as waste, as a highly clinically promising biological product due to its content of various bioactive factors. The collection of these factors is often referred to as mesenchymal stem cell metabolites (MSCM). 9 The proteomic analysis of MSCM has reported that it contains a variety of growth factors, cytokines, chemokines, and interventional RNA (iRNA). Bioactive molecules contained in MSCM have been postulated to interfere with multiple biological activities positively, especially wound healing and tissue regeneration. 9 10 11 12 During the wound healing process, the factors in MSCM can interact with host tissue via the paracrine mechanism, activating several pro-regenerative pathways. 10 Another study reported that metabolites of mesenchymal stem cells (MSCM) are material with good biocompatibility and can induce the proliferation of human gingival somatic cells in *in vitro* studies. 13 These findings initiate the development of adipose mesenchymal stem cell metabolites (AdMSCM) oral gel as the mean of MSCM application in the oral mucosa. This study will confirm whether the application of AdMSCM oral gel can enhance the expression of VEGFA and FGF-2, angiogenesis, and clinical outcome in oral ulcer rat models.

## Materials and Methods

### Study Design and Setting

Faculty of Dental Medicine, Universitas Airlangga, Surabaya, Indonesia assigned this study protocol ethical approval for animal laboratories with number 209/HRECC.FODM/IV/2022. This work is a true experimental laboratory study using an analytical post-test control group design. Lemeshow's formula determines the minimum sample; the total sample needed is 20, with five samples for each group. Male Wistar rats ( *Rattus norvegicus* ) weighing 250–300 g and 1 to 2 months old made up the sample. The experimental animals were free of any oral and systemic pathologies.

### AdMSCM Oral Gel Preparation

The AdMSCM oral gel was acquired from a patent owned by Research Center for Vaccine Technology and Development, Institute of Tropical Disease, Universitas Airlangga, Surabaya, Indonesia. The AdMSCM Gel preparation begins with the collection of plain culture medium after the fourth passage of AdMSC culture. Then, the AdMSC culture medium was purified using the dialysis method to remove the remnants of metabolic products, resulting in the isolated soluble bioactive factors released by AdMSC during culture. The purified culture media was combined with 5% hydroxypropyl-methylcellulose (HPMC) with a 1:3 volume ratio to create AdMSCM oral gel with 30 mg/mL concentration.

### Oral Ulcer (OU) Animal Model

An oral ulcer was created using an 8 g/3 mm punch biopsy tool (Premier, Plymouth, USA) on the mucosa of the inferior incisive labial fornix. Then, the base of the tissue was cut using a no. 15 surgical blade. 14 The lesion was then clinically observed 24 hours after punch-biopsy to assess the formation of OU (clinically characterized by a white-colored lesion surrounded by an erythematous arc). While making traumatic ulcers, experimental animals were under general anesthesia using sodium pentobarbital which was injected intramuscularly into the gluteal region.

The oral ulcer treatment in animals was divided into four groups, and they were treated with 5% HPMC (control) and AdMSCM oral gel. The treatment was performed three times daily, using disposable micro applicators (Cotisen, China), for 3 and 7 days. The termination of experimental animals was performed using the cervical dislocation technique. Before termination, the animals were anaesthetized using a single intraperitoneal injection of pentobarbital 50 mg/kg (pentobarbital solution, no. cat: P-010, Sigma Aldrich) systemically (20–40 mL).

### Clinical Evaluation of Ulcer's Diameter

The major ulcer diameters of each sample were measured using a fine precision ruler (Fisherbrand, Pittsburg, USA) with a 1 mm increment. The measurement was conducted while the animals were under general anesthesia.

### Tissue Preparation

An excisional biopsy of the inferior fornix labial mucosa was conducted for tissue harvesting. The tissues were then fixated in a 10% neutral buffer formalin (NBF) solution (Sigma Aldrich, California, USA) for three days. After that, the tissues were washed three more times with PBS (OneMed, Sidoarjo, Indonesia) for 5 to 10 minutes each. Then, the tissues were embedded in paraffin and sectioned with a rotary microtome to obtain HPA slides.

### Histological Evaluation of Angiogenesis

The histopathology slides were processed and stained with hematoxylin–eosin (HE). Then, the angiogenesis of each slide was observed using a light microscope with 100 × , 400 × , and 1000× magnification (Nikon, H600L, Japan) at the Dental Research Center, Faculty of Dental Medicine, Universitas Airlangga. The calculations of the angiogenesis number were performed in five different fields of view by two observers.

### Immunohistochemistry

The immunohistochemistry staining was used to evaluate VEGFA and FGF-2 positive expressions in the tissues. The histopathology slides were processed immunohistochemically using horse radish-labeled monoclonal antibodies (anti-VEGFA #SC-7269 ( *Santa Cruz Biotechnology* Inc., California, USA), FGF-2 #SC-74412 ( *Santa Cruz Biotechnology* Inc., California, USA)), and 3–3′ diaminobenzidine (DAB) (Abcam, USA). Then, the positive expression of the protein marked by brown precipitate on the cells of the oral ulcer site was observed using an inverted light microscope with 100 × , 400 × , and 1000× magnification in five different fields of view by two observers.

### Statistical Analysis

Statistical analysis was performed using GraphPad Prism 8.0 ( for MacBook, v9.4.1, San Diego, USA). The normality test was performed using the Shapiro–Wilk test and the homogeneity test using Levene's test. If the data obtained met the requirements for parametric tests, a one-way analysis of variance (ANOVA) test ( *p* < 0.05) is going to be conducted to find the differences between all groups. If the data did not meet the requirements for a parametric test, a nonparametric test was performed using the Kruskal–Wallis test ( *p* < 0.05). Then, the multiple comparison post-hoc test using Tukey's HSD was performed to uncover specific differences between groups.

## Results

In this investigation, it was discovered that the data on the ulcer diameter, angiogenesis, and the expression of VEGFA and FGF-2 was homogeneous and fitted with Gaussian distribution ( *p* > 0.05); thus, comparative parametric analysis using one-way ANOVA was performed with statistical significance is assumed at the *p* < 0.05.

### Clinical Outcome

**Fig. 1:**
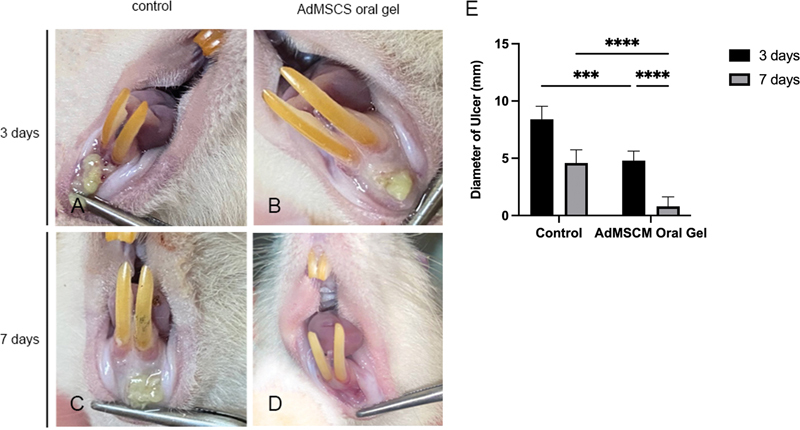
The clinical of oral ulcer in animals. ( **A** ) control after three days of treatment; ( **B** ) AdMSCS oral gel after three days of treatment; ( **C** ) control after seven days of treatment; ( **D** ) AdMSCS oral gel after seven days of treatment and ( **E** ) The AdMSCS oral gel treatment significantly increased the oral ulcer healing. *** *p* < 0.001; **** *p* < 0.0000

Clinical images showing the oral ulcers after treatment for 3 and 7 days can be seen in Fig. 1A-1D . The AdMSCM oral gel treatment groups reveals substantial evidence of a significant reduction in ulcer diameter found after treatment for three ( *p* = 0.0002) and seven days ( *p* < 0.0001) ( Fig. 1E ; Table 1 ).

**Table 1:** Ulcer diameter, angiogenesis, and the expression of FGF-2 and VEGFA in an oral ulcer rat model

Treatment Duration	Group	Ulcer Diameter	Angiogenesis	VEGFA	FGF-2
		Mean ± SD					
Three days	control	8.4 ± 1.140	4.8 ± 1.304	2.2 ± 0.837	2.2 ± 0.837
	AdMSCM oral gel	4.6 ± 1.140	4.2 ± 1.304	4.0 ± 1.000	4.8 ± 1.304
Seven days	control	4.8 ± 0.837	5.2 ± 0.837	7.0 ± 1.581	7.0 ± 1.581
	AdMSCM oral gel	0.8 ± 0.837	9.0 ± 1.581	10.0 ± 1.581	11.40 ± 1.140
*p* -Value		0.0001*	0.0001*	0.0001*	0.0001 *

Abbreviations: AdMSCM, adipose mesenchymal stem cell metabolite; FGF-2, fibroblast growth factor-2; OU, oral ulcer; SD, standard deviation. VEGFA, vascular endothelial growth factor A.

* Significant difference of one-way ANOVA represented by *p* < 0.05.

### Angiogenesis

**Fig. 2:**
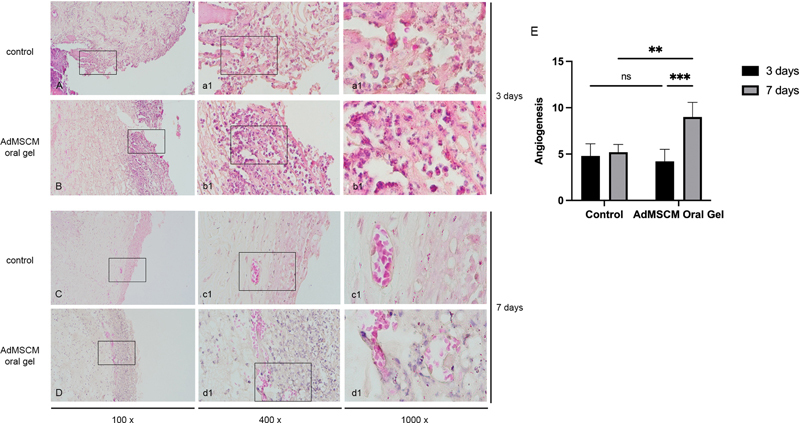
The histopathology of oral ulcer with hematoxyline eosin staining in control and AdMSCM oral gel treatment for three days ( **A and B** ) and seven days ( **C and D** ). The AdMSCM oral gel treatment increased the angiogenesis ( **E** ). ** *p* < 0.01; *** *p* < 0.001; ns: not significant

The histologic assessment of angiogenesis in the oral ulcer site is represented in Fig. 2A-2D . The AdMSCM oral gel treatment on oral ulcers for three days did not significantly increase the angiogenesis ( *p* = 0.9597). However, significantly higher angiogenesis was found seven days after AdMSCM oral gel treatment ( *p* = 0.0001; Fig. 2E ; Table 1 ).

### FGF-2 Expression

**Fig. 3:**
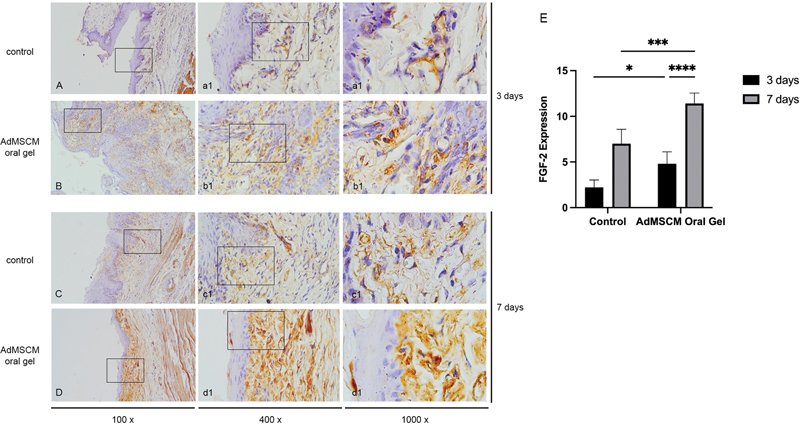
The histopathology of oral ulcer with immunohistochemistry staining in control and AdMSCM oral gel treatment for three days ( **A and B** ) and seven days ( **C and D** ). The AdMSCM oral gel treatment increased the FGF-2 expression ( **E** ). * *p* < 0.05; *** *p* < 0.001; **** *p* < 0.0000

The histopathology view of the FGF-2 expression pattern in the oral ulcer site can be seen in Fig. 3A-3D . Brown precipitates in the epithelial layer and connective tissue of the ulcer site mark FGF-2 expression. According to the results of the one-way ANOVA, there were statistically significant differences between all groups ( *p* = 0.0001; Table 1 ). The AdMSCS oral gel demonstrates a significantly higher FGF-2 expression after three ( *p* < 0.0001) and seven days ( *p* < 0.0001) compared with the control ( Fig. 3E ).

### VEGFA Expression

**Fig. 4:**
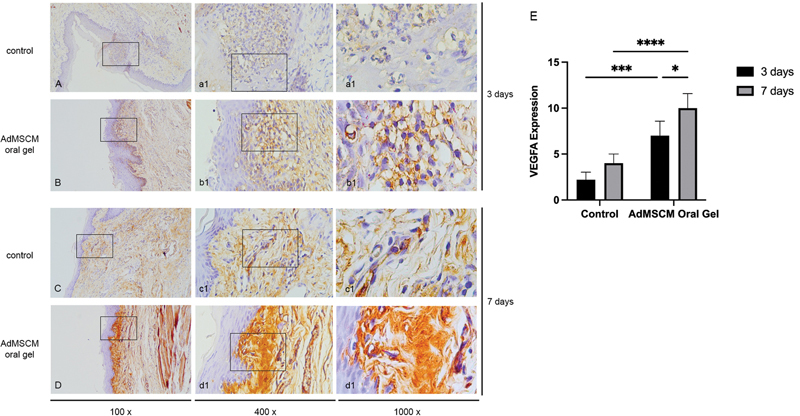
The histopathology of oral ulcer with immunohistochemistry staining in control and AdMSCM oral gel treatment for three days ( **A and B** ) and seven days ( **C and D** ). The AdMSCM oral gel treatment increased the VEGFA expression after seven days of treatment ( **E** ). * *p* < 0.05; *** *p* < 0.001; **** *p* < 0.0000

The VEGFA expression pattern is marked by brown precipitates in the epithelial layer and connective tissue of the ulcer site, as seen in Figure 4A-4D. The VEGFA expression after AdMSCM oral gel treatment for 3 and 7 days was higher than the control ( *p* = 0.0001 and *p* < 0.0001, respectively; Fig. 4E ).

## Discussion

AdMSCM oral gel contains a collection of bioactive molecules, such as growth factors, cytokines, chemokines, and noncoding RNAs (e.g., micro RNA [miRNA]) produced by MSCs during culture. The study by Park et al reported that AdMSCM contains more than 40 types of growth factors, mainly EGF, FGF-2, IGF-1, and HGF, which are reported to be able to accelerate the wound healing process via a paracrine mechanism. 9 10 The same study also reported that the administration of AdMSCM was able to increase the activity of the PI3K/Akt or FAK/ERK1/2 pathways that play a role in regulating the proliferation and migration activity of various skin and oral mucosal cells, such as fibroblasts, keratinocytes, and vascular endothelial cells that will induce the process of wound contraction. 10 In addition, another study by Nugraha et al reported that MSCM is biocompatible, nontoxic, and able to increase the proliferation of human somatic cell culture 12 hours after application. 13 These studies supported the finding of this study that reported the excellent clinical outcome of the AdMSCM oral gel treatment groups.

Limiting inflammatory activity in wound healing is essential for creating an optimal healing process. 15 16 Previous studies reported that a prolonged inflammatory phase of wound healing would cause the proliferative phase not to be achieved, which may lead to the formation of chronic wounds. 2 5 In addition to proregenerative effects, AdMSCM oral gel can exert immunomodulatory effects produced by various anti-inflammatory components. 11 AdMSCM contained various soluble anti-inflammatory cytokines (such as TGF-β, IL-10, and PGE-2), extracellular proteins that act as a resolution-associated molecular pattern (RAMPs) (e.g., IDO, HSP10, HSP70), and interference RNAs (e.g., the miR-family). 17 18 19 According to research by Zriek et al, the immunomodulatory components of MSCM can interact with immune cells to activate anti-inflammatory regulatory phenotypes. 20

Macrophages with the M1 phenotype are one of the primary mediators of inflammation in wound healing. Various anti-inflammatory cytokines (e.g., TGF-β and IL-10) present in the AdMSCM oral gel can interact with their respective receptors on macrophages and trigger the polarization of pro-inflammatory macrophages (M1) to anti-inflammatory macrophages (M2). In addition, anti-inflammatory cytokines can also induce the activation of M0 macrophages through the alternative pathways. This theory is proven by Holthauss et al where it was reported that the administration of preconditioned AdMSCM to M0 cells was able to increase the expression of Arg1 and MerTK genes, which are genes that are highly expressed in the activation of the alternative pathway of macrophages (M0 to M2 polarization). 18 The role of RAMPs in the resolution of inflammation was also demonstrated in the study by Borges et al, where HSP70 can produce an anti-inflammatory effect through the activation of the TLR-2 pathway, which will trigger the activation of downstream ERK proteins to induce the production of IL-10. 21 Recent studies have also demonstrated the critical role of miRNAs in the resolution of inflammation in physiological wound healing. 22 23 The immunomodulatory effect of miRNA is evidenced by the reported role of miR-let7b in triggering M1 to M2 polarization. 24 Accelerated resolution of inflammation in the wound healing process can accelerate the transition to the proliferative phase so that it will indirectly accelerate wound closure, as illustrated in the results of this study. 23 In addition, miRNAs have also been reported to regulate the proliferative phase of wound healing, specifically at the stages of re-epithelialization, angiogenesis, and granulation tissue formation. 22

This study also reported that the level of angiogenesis that occurred in the treatment group was significantly higher than in the control group ( *p* < 0.05). Angiogenesis is the growth of new capillaries from existing blood vessels. 4 This event occurs in the proliferative phase of the wound-healing process. 2 This process is crucial to creating an effective and optimal wound-healing process. 4 The process of angiogenesis is initiated by an injury that causes microvascular endothelial cells (MEC) that line the tunica intima of blood vessels to be activated by hypoxia and pro-angiogenic factors such as VEGF and FGF-2. 25 Angiogenesis primary function in wound healing is to facilitate oxygen and nutrient supply and transport cells and molecules. The content of pro-angiogenic factors (e.g., VEGF and FGF-2) in the AdMSCM oral gel is thought to have contributed to the findings in this study. Previous study reported similar results, where the application of AdMSCM could significantly increase the expression of CD-31 (platelet endothelial cluster adhesion molecule-1/PECAM1), a specific marker molecule of vascular endothelial cells. 10 Angiogenesis can also be induced by miRNAs contained in MSCM. 19 Previous studies reported that MSCM contains several pro-angiogenic miRNAs, such as miRNA-23a. 19 miRNA-23a can inhibit the prolyl hydroxylase ½ (PHD ½) gene, leading to the accumulation of HIF-1α in vascular endothelial cells, thereby inducing angiogenesis. 26 In addition, miRNA-23a can increase vascular permeability and cell migration which is crucial in angiogenesis. 22

As the primary pro-angiogenic factor, observing VEGFA expression may indicate the potency of AdMSCM oral gel in enhancing angiogenesis. 4 This study also found that the expression of VEGFA in the AdMSCM oral gel treatment group on the third and seventh days was significantly higher than in the control group ( *p* < 0.05). A similar result was also reported by Sunarto et al, where the administration of MSCM can increase VEGF levels in skin wound model. 27 In addition, similar results were also found in the observation of FGF-2 expression, where the mean FGF-2 expression in the AdMSCM oral gel treatment group on the third and seventh days was significantly higher than the control group ( *p* < 0.05). The importance of FGF-2 in wound healing is not only limited to their function to induce angiogenesis, but also the proliferation of fibroblast in granulation tissue formation phase. FGF-2 able to induce proliferation of human dermal fibroblast via the activation of ERK ½ and JNK pathways. 28 29 The findings that the expression of these two growth factors in the treatment group was significantly higher than in the control group can be explained by the significant role of M2 macrophages in producing various growth factors that can induce wound healing. 2 30 Larjava et al reported that M2 macrophages could produce various growth factors relevant to the wound healing process, such as VEGFA, IGF-1, FGF-2, and HGF. 2 31 As previously mentioned, the immunomodulatory content of the AdMSCM oral gel was able to induce pro-reparative M1 to M2 polarization of macrophages. 30 32

## Conclusion

This investigation reveals that the topical application of AdMSCM oral gel is able to enhance the clinical outcome, angiogenesis, and expression of VEGFA and FGF-2 in the oral ulcer animal model ( *R. novergicus* ). Further exploration through other approaches is still required to evaluate AdMSCM oral gel potency in inducing oral ulcer healing.
